# Enhancing mental and physical health of women through engagement and retention (EMPOWER) 3.0 QUERI: study protocol for a cluster-randomized hybrid type 3 implementation-effectiveness trial

**DOI:** 10.1186/s13012-026-01521-3

**Published:** 2026-07-08

**Authors:** Alison B. Hamilton, Melissa M. Farmer, Bevanne Bean-Mayberry, Tannaz Moin, Ariel Lang, Lynette Adams, Catherine Chanfreau, Karissa Fenwick, Ismelda Canelo, Rebecca Oberman, Erin P. Finley, EMPOWER 3.0 QUERI Team

**Affiliations:** 1 Veterans Affairs Health Systems Research (HSR) Center for the Study of Healthcare Innovation, Implementation & Policy, VA Greater Los Angeles Healthcare System, Los Angeles, CA USA; 2 Department of Psychiatry and Biobehavioral Sciences, David Geffen School of Medicine, University of California Los Angeles, Los Angeles, CA USA; 3 Division of General Internal Medicine and Health Services Research, Department of Medicine, David Geffen School of Medicine, University of California Los Angeles, Los Angeles, CA USA; 4 Division of Endocrinology, Diabetes & Metabolism, Department of Medicine, David Geffen School of Medicine, University of California Los Angeles, Los Angeles, CA USA; 5 VA San Diego Healthcare System, San Diego, CA USA; 6 Department of Psychiatry, University of California San Diego, San Diego, CA USA; 7 District of Columbia, U.S. Department of Veterans Affairs, Office of Women’s Health, Washington, USA; 8 Departments of Medicine and Psychiatry and Behavioral Sciences, University of Texas Health Science Center, San Antonio, TX USA

**Keywords:** Hybrid effectiveness-implementation, Cluster randomized trial, Women Veterans, Mixed methods, Co-production, Implementation strategies, Evidence-based quality improvement, Replicating effective programs, Trauma-informed care, Menopause and perimenopause, Lifestyle and weight management

## Abstract

**Background:**

Women Veterans are the fastest growing segment of VA users, with most users in midlife. This dramatic growth has challenged VA to ensure that appropriate services are available to meet women Veterans’ needs, and that they are willing and able to use those services. Furthermore, few VA improvement efforts have focused on women Veterans in midlife. Enhancing Mental and Physical Health of Women through Engagement and Retention (EMPOWER) 3.0 builds on prior work to optimize women Veterans’ access to and quality and experience of care. It offers trauma-informed, evidence-based interventions that meet high-priority health needs in midlife, with the objective to increase women Veterans’ trust, well-being, and engagement in VA care.

**Methods:**

EMPOWER 3.0 will evaluate two implementation strategies, an Evidence-Based Quality Improvement booster (EBQI/B) and EBQI/B plus external facilitation (EBQI/B+EF). These strategies are designed to support implementation of three evidence-based practices (EBPs) for women Veterans: Trauma-Informed Care, a VA-mandated set of activities and approaches that support trust and safety in primary care interactions; Cognitive Behavioral Therapy for Symptoms of Menopause, which provides group-based therapy, education, and personalized referrals for women across the menopausal transition; and Women’s MOVE!, which provides tailored lifestyle and weight management support to enhance women’s health and well-being. Year 1 activities will include iterative co-production to tailor each intervention to meet the needs of women Veterans and diverse VA sites. We will conduct a mixed methods implementation evaluation using a cluster-randomized hybrid type 3 effectiveness-implementation trial design to compare the effectiveness of EBQI/B and EBQI/B+EF in terms of: (a) access to trauma-informed care tailored for women Veterans; (b) implementation strategy fidelity, speed of implementation, and sustainment; (c) recipient perspectives on and experiences of EBP implementation; and (d) costs of implementation and business case analysis to support sustained EBP use.

**Discussion:**

Grounded in the Health Equity Implementation Framework, EMPOWER 3.0 provides an innovative model for conducting partnered and collaborative mixed methods implementation in a hybrid type 3 effectiveness-implementation project; the evaluation will comprise a comprehensive set of measures including performance metrics, implementation progress, stakeholder experience, and cost and return on investment across a complex organizational setting.

**Supplementary Information:**

The online version contains supplementary material available at https://doi.org/10.1186/s13012-026-01521-3.

## Contributions to the literature

• Few studies have compared the effectiveness of implementation strategies for achieving rollout of multiple evidence-based interventions across large healthcare systems.

• Conducting rigorous comparison of clinical benefit, cost and return on investment, and time to achieve implementation associated with two different implementation strategies – an Evidence-Based Quality Improvement booster (EBQI/B) and EBQI/B plus external facilitation – can directly support evidence-based policy and resource allocation decisions.

• This study protocol provides an innovative model for conducting partnered and collaborative mixed method implementation evaluation in a national healthcare system.

## Background

The number of women Veterans enrolled in VA healthcare services has increased dramatically, from 159,801 in 2001 to over 650,000 currently [1]. Nearly half are in midlife (aged 45–64), with a median age of 48 years [2]. A growing number of studies [3–5] identify gaps in health care for women Veterans in this midlife stage, during which menopause typically occurs, 49% of women Veterans meet criteria for obesity [6], and the risk of chronic disease increases dramatically. Furthermore, women Veterans have a high burden of trauma and physical and mental health comorbidities, which may exacerbate menopause symptoms [7] and chronic disease risk [8] as well as negatively impact healthcare engagement and trust. Notably, only 40% of women in VA care report “complete trust” in their VA provider [9]. Lack of trust in VA can drive seeking community care [10], which is associated with lower quality of care, higher costs, and eventual attrition from VA [11]. While VA has invested substantially in improving health care for women Veterans, continued improvement efforts are needed to ensure accessible, effective, trustworthy, and trauma-informed care for women Veterans, with concerted efforts for women in midlife.

Since its inception in 2015, the Enhancing Mental and Physical health of Women through Engagement and Retention (EMPOWER) QUERI team has been improving access to evidence-based practices (EBPs) in women’s health [12, 13]. Across EMPOWER 1.0 and 2.0, we have implemented EBPs that improved women’s access to and utilization of care for mental health, cardiovascular health, and type 2 diabetes prevention, by tailoring virtual interventions [14–23]. In the current study protocol, our EMPOWER 3.0 Impact Goal is to optimize women Veterans’ access to and quality and experience of care by offering trauma-informed, evidence-based interventions that meet high-priority health needs in midlife, and thereby, to increase women Veterans’ trust, well-being, and engagement in VA care. This Impact Goal is guided by input from our longstanding VA operations partners: the Offices of Women’s Health (OWH), Mental Health (OMH), Patient-Centered Care and Cultural Transformation (OPCC&CT), Rural Health (ORH), and the National Center for Health Promotion and Disease Prevention (NCP). To support sites with implementation, we will leverage a multi-year, multi-million dollar investment made by VA OWH in rolling out Evidence-Based Quality Improvement (EBQI) across VA women’s health [24]. EBQI has demonstrated effectiveness in VA women’s health, contributing to improved women’s primary and specialty care [25, 26]. In collaboration with OWH, our team builds upon a unique opportunity to assess the additional supports needed to successfully launch novel EBPs in women’s health clinics across four partnering Veteran Integrated Service Networks (VISNs).

**Fig. 1:**
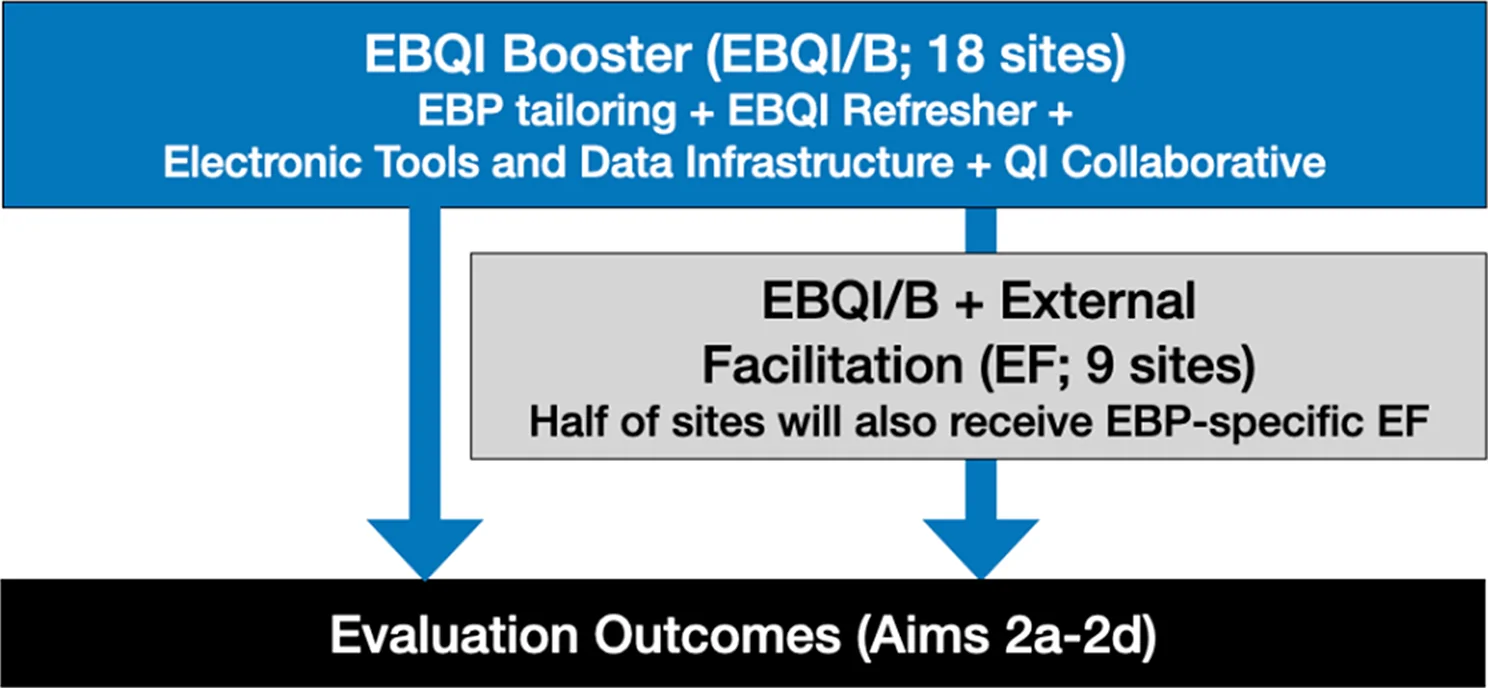
EMPOWER 3.0 Implementation Strategies

To achieve this impact goal, we established a coordinated program with the following aims:

1. Support implementation and sustainment of three EBPs for women Veterans in midlife using an EBQI Booster (EBQI/B) strategy in 18 VA facilities that have completed the OWH EBQI initiative. Half of these sites (*n* = 9) will be randomized to *also* receive 12 months of external facilitation (EF): EBQI/B + EF (Fig. 1).
2. Conduct a multi-method implementation evaluation using a type 3 hybrid implementation-effectiveness cluster-randomized trial design. We will compare effectiveness of EBQI/B and EBQI/B + EF in terms of: (a) improved access to care that is trauma-informed and tailored for women Veterans; (b) implementation strategy fidelity, speed of implementation, and sustainment; (c) recipient perspectives on and experiences of EBP implementation; and (d) costs of implementation and business case analysis to support sustained EBP use.
3. Develop a Translation-to-Policy (T2P)-informed scale-up and evaluation plan [27] to support sustainment, spread, and continuous improvement of trauma-informed EBPs in VA care.

These conceptually and methodologically linked aims will be realized by an experienced multidisciplinary team with a strong track record of collaboration and guided by a Learning Community of operational and research partners and subject matter experts. We will implement three EBPs: (1) Trauma-Informed Care (TIC), a VA-mandated [28] set of activities and approaches that support trust and safety in primary care interactions; (2) Cognitive Behavioral Therapy for Symptoms of Menopause (CBT-SM), which provides group-based therapy, education, and personalized referrals for women across the menopausal transition; and (3) Women’s MOVE! (Managing Overweight and Obesity for Veterans Everywhere), which provides lifestyle and weight management support tailored for women Veterans. (For detailed background on these EBPs and related health conditions among women Veterans, see Appendix 1.)

## Methods/design

### Overview of study design

This five-year type 3 hybrid [29, 30] implementation-effectiveness cluster-randomized trial will compare the effectiveness of two implementation strategies (EBQI/B vs. EBQI/B + EF) for supporting implementation of three EBPs to improve access to, quality of, and engagement in care for women Veterans in midlife (Aim 1). Aim 2 involves a multi-method implementation evaluation aligned with QUERI and operations partners’ priorities. Aim 3 will collaborate with the EMPOWER 3.0 Learning Community to develop scale-up and evaluation plans informed by the Translation-to-Policy (T2P) Learning Cycle [27], creating a roadmap for sustaining and spreading improved care practices for women Veterans. We describe our three Aims in detail below.

### Cluster-randomized trial of the implementation strategies

**Fig. 2:**
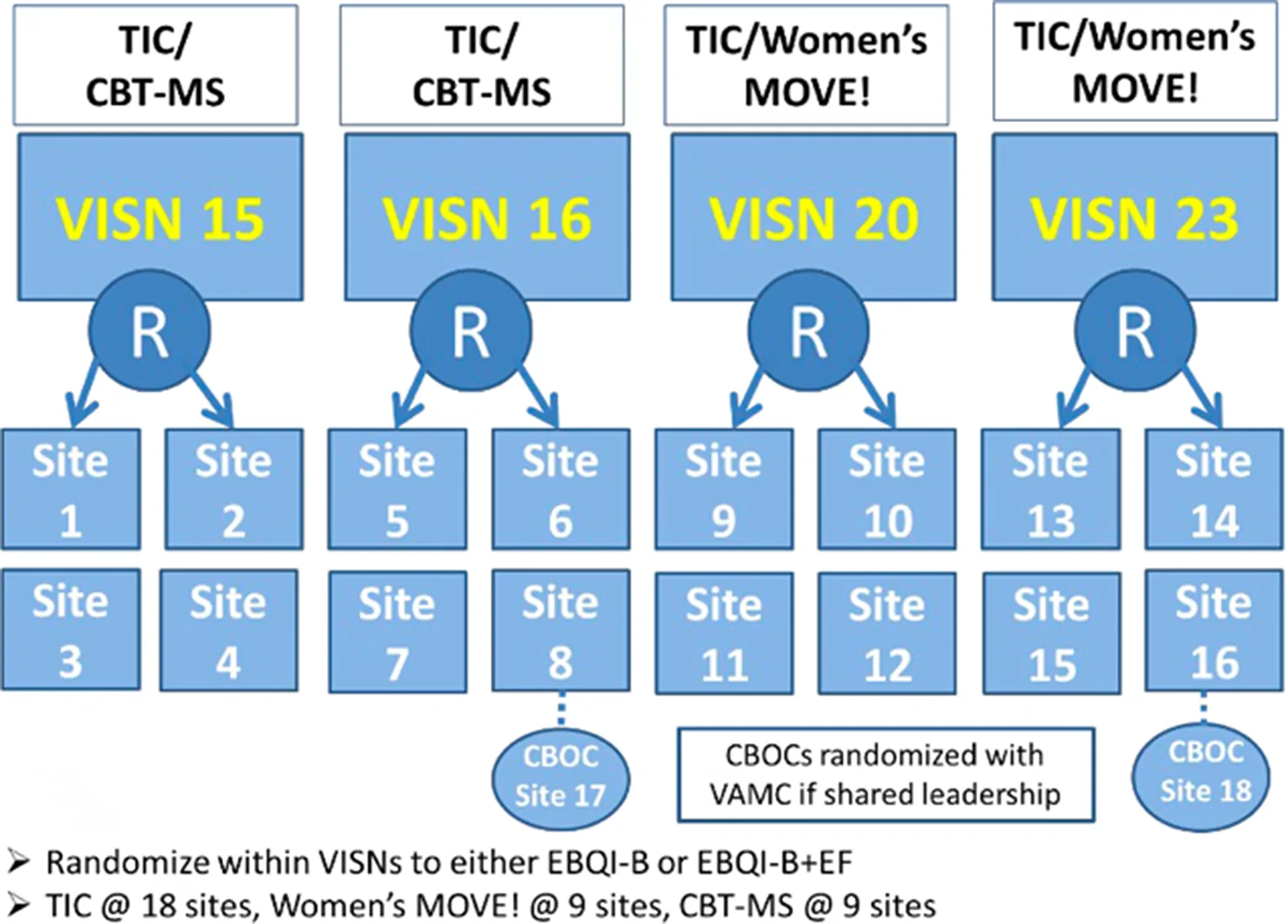
Site-Level Randomization (Sites = 18)

For the cluster-randomized trial, we will randomize sites to either EBQI/B or EBQI/B + EF within each of four Veteran Integrated Service Networks (VISNs), with half of sites randomized to EBQI/B and half to EBQI/B + EF (Fig. 2). In response to high demand, all sites will have the option to implement TIC in primary care settings. In addition, sites from two VISNs will implement CBT-SM and sites from two VISNs will implement Women’s MOVE! (Fig. 2). After the study, all sites will be provided with finalized T2P scale up and evaluation plans to ensure access to all EBPs. We are cognizant that the same WH leadership team (e.g., WH Medical Director, WV Program Manager) may oversee both VA Medical Center (VAMC) and affiliated Community-Based Outpatient Clinic (CBOC) WH operations; these will therefore be randomized as a unit to avoid contamination across strategies.

We have recruited VISNs and sites in collaboration with the Women’s Health Practice-Based Research Network (WH-PBRN), a network that provides research infrastructure for investigators conducting multi-site women’s focused research in VA [31]. The WH-PBRN facilitated initial site recruitment via email to Site and/or VISN Leads, after which our team met with the Leads to discuss the opportunity and provide additional information materials.

### Aim 1. Support implementation and sustainment of three EBPs for women Veterans in midlife

### Implementation strategies

*EBQI in VA women’s health*. With demonstrated effectiveness in multiple VA implementation trials [26, 32], EBQI is a systematic QI method for engaging frontline practices in improvement that introduces multilevel engagement, “best science” and evidence in the service of operational goals [33]. EBQI is aimed at developing learning organizations, drawing upon use of QI methods to enable context-tailored EBPs and social science theory on provider/team behavior. In VA women’s health, EBQI was first tested in a 12-site cluster-randomized trial examining tailoring VA’s Patient-Aligned Care Teams (PACT) to meet women Veterans’ needs [34]. Given robust mixed methods findings (e.g., improved team functioning) [26, 35], OWH invested in further application of EBQI in 21 low-performing sites; in a dynamic wait-listed design, EBQI was rolled out in women’s health over the course of three years, with a contractor delivering EBQI and the research team conducting the evaluation [24]. OWH later launched the *Women Veterans Achieving Comprehensive Health Innovation and Enhancement for Women Veterans through EBQI (WV-ACHIEVE)* initiative, with the goal of contractors helping facilities to “improve delivery and quality of comprehensive care, services, and/or experiences for women Veterans” [36]. Participating sites reported improved relationships with leadership and other service lines, increased motivation to pursue process change, and improved knowledge of best practices for QI and change management [37]. However, they also reported need for additional capacity-building, including through continued access to EBQI training and materials, as well as through guidance on initiating future projects without support from the contractors. To promote sustainment of EBQI in women’s health, OWH encouraged our team to conduct EMPOWER 3.0 at sites that completed WV-ACHIEVE in FY21-25.

*Implementation Mapping*. EMPOWER 3.0 builds on a remarkable opportunity to enhance the OWH investment in EBQI by testing what level of additional support is needed by WV-ACHIEVE sites to successfully launch novel EBPs, including whether a low-touch (EBQI/B) or high-touch (EBQI/B + EF) strategy is required. In selecting these strategies, we have worked collaboratively through an implementation mapping [38] process with our sites, the WH-PBRN, and our operations partners, by: (1) conducting a needs and assets assessment [39] and identifying potential implementers for each of the proposed EBPs; (2) identifying adoption and implementation outcomes, performance objectives and determinants, and developing an *Implementation Research Logic Model* [40]; (3) selecting implementation strategies well-suited to address existing barriers (e.g., inconsistent training across WH clinical teams in trauma-informed care); (4) developing a comprehensive implementation plan (Aim 1); and (5) preparing evaluation plans that will, via both the proposed hybrid trial (Aim 2) and longer-term evaluation plans to be developed in Aim 3, deliver actionable evidence to inform VA policy decision-making and continuous improvement efforts.

*EBQI Booster (EBQI/B).* Our EBQI/B strategy bundle comprises four components that will be delivered to all sites and will begin during the pre-implementation period (see Table 1).

- *Co-Production* (Year 1). Building on our needs and assets assessment and lessons from EMPOWER 1.0 and 2.0 [14, 15, 17, 19], and addressing critiques of limited patient engagement in EBQI [41], we will co-produce all three EBPs with Veterans and the Learning Community. Consistent with ADAPT guidance [42], this collaborative process will ensure alignment between interventions and the needs of women Veterans, VA providers, and diverse VA sites while maintaining intervention integrity. Co-production involves engaging all stakeholders to optimize EBP packaging and delivery, emphasizing shared power, diverse perspectives, and reciprocity—critical for meeting the needs of diverse women Veterans [43]. Preliminary feedback from operations partners and the VA Women’s Improvement Network (WIN; October 2024) informed our EBP selection. We will work directly with the Learning Community and conduct semi-structured interviews with women Veterans to refine EBP packaging and delivery.
- *EBQI Booster*. Given that WV-ACHIEVE sites vary in their time since exposure to the initiative, may have experienced staff turnover, and frequently request additional capacity-building [37], we will provide each site with a two-hour EBQI Booster delivered virtually to site-identified QI champions and local implementation teams for each EBP. This Booster will review key EBQI components and provide examples grounded in the 3.0 EBPs.
- *Electronic implementation tools and data infrastructure*. For EMPOWER 2.0, we developed user-friendly electronic implementation tools including quality dashboards that integrated EHR data from VA Corporate Data Warehouse (CDW), women-focused VA performance metrics, and EBP program-specific data, as well as a SharePoint to facilitate EBP recruitment at the local sites [13]. These dashboards will serve as a foundation for new and enhanced tools in EMPOWER 3.0. *Quality dashboards*: We will automate data integration from CDW/FEHR and other data sources, creating dashboards in the enterprise cloud using PowerBI. Dashboards will provide users with dynamic reports to visualize site-level cohorts and performance gaps, and drill down on subgroups (e.g., age cohorts, rurality, race/ethnicity) to identify individuals for recruitment and intervention. They will include patient-panel characteristics, weight/body mass index (BMI) trends, women-specific VA performance metrics (e.g., well-being, trust), and Whole Health utilization (using OPCC&CT algorithm), and support implementation monitoring of each EBP. *Recruitment tool*: We will provide a site-level SharePoint to support patient recruitment for the EBPs. The tool will apply algorithms to identify eligible patients using EHR data, stratify by demographics (e.g., rurality, race/ethnicity) and risks (e.g., obesity status, diagnosis, laboratory test results such as A1c level), generate patient contact lists, and track outreach using a write-back function. *New tools*: Guided by the Learning Community, the WIN, and usability testing with providers and patients, we will leverage Annie, an existing VA patient- and provider-facing app [44], to program automated self-care reminders/texts tailored to each EBP. We will use electronic interfaces to collect patient-reported outcomes (PROs, e.g., trust, satisfaction, physical activity) and feedback using short surveys sent via REDCap [45]. For Women’s MOVE!, weight information from wireless scales will be tracked via Patient-Generated Health Data (PGHD) [46]. Data will be integrated into dashboards to provide actionable site feedback. Participating site teams will be trained in use of the tools to support information-gathering and evaluation relevant to EBP implementation and evaluation, consistent with EBQI principles for rigorous use of data to drive improvement efforts [47].
- *QI Collaboratives*. QI collaboratives are a core element of EBQI [47] and provide an ongoing opportunity for cross-site learning and knowledge-sharing, targeting implementation mechanisms for enhancing EBP knowledge/skills and collaborative, data-driven problem-solving. We will offer EBP-specific QI Collaboratives that include a Teams channel, on-demand technical assistance from the EMPOWER 3.0 Implementation Support teams, and quarterly QI Collaborative calls to report on relevant policy and practice updates and share knowledge around challenges and successes. Separate QI Collaborative calls for EBQI/B and EBQI/B + EF sites will avoid contamination across the two conditions.

*EBQI/B + External Facilitation (EF)*. A recent systematic review of experimental studies of implementation strategies identified EF as one of the most-studied strategies (tested in 59 studies), associated with positive results in at least 75% of relevant studies [48]. EF, delivered by an expert facilitator from outside the local implementation site, offers a supportive coaching process in which facilitators encourage interactive problem-solving in improvement efforts [49]. In delivering EF as part of EBQI in the EMPOWER 2.0 trial, our team has developed refined workflows and processes for ensuring high-fidelity delivery and documentation, including templated call sheets to guide monthly calls with participating sites [50]. Our experienced team of external facilitators will update and apply these processes in EMPOWER 3.0; sites randomized to the EBQI/B + EF condition will receive 12 months of monthly EF virtual conference calls, beginning in Year 2.

**Table 1:** Implementation strategies by QUERI implementation roadmap phase [51]

	Year 1	Years 2–4	Year 5
QUERI RoadmapPhase	Pre-implementation	Implementation	Sustainment
EBQI Booster (EBQI/B)	• Refine intervention packages via *Co-production* with women Veterans and EMPOWER 3.0 Learning Community • Provide *EBQI Refresher* training for site QI champions and teams • Train sites in use of *Electronic Implementation Tools* • Convene cross-site EBP-specific *QI Collaboratives*	• Continue QI Collaboratives (via Teams channel, consult on-demand, and quarterly cross-site calls)	• In collaboration with the Learning Community, develop Translation-to-Policy (T2P)[27]-informed scale-up and evaluation plans to include revised EBP packages and implementation playbooks
EBQI/B + External Facilitation	• As above.	• As above. Sites randomized to this condition also receive 12 months of monthly EF calls, to include expert review and feedback on implementation challenges and progress.	• As above.

EBQI: Evidence-Based Quality Improvement; B: Booster; QI: Quality Improvement; EBP: Evidence-Based Practice; EF: External Facilitation

### Aim 2. Conduct a*multi-method implementation evaluation*using a type 3 hybrid implementation-effectiveness cluster-randomized trial design

**Fig. 3:**
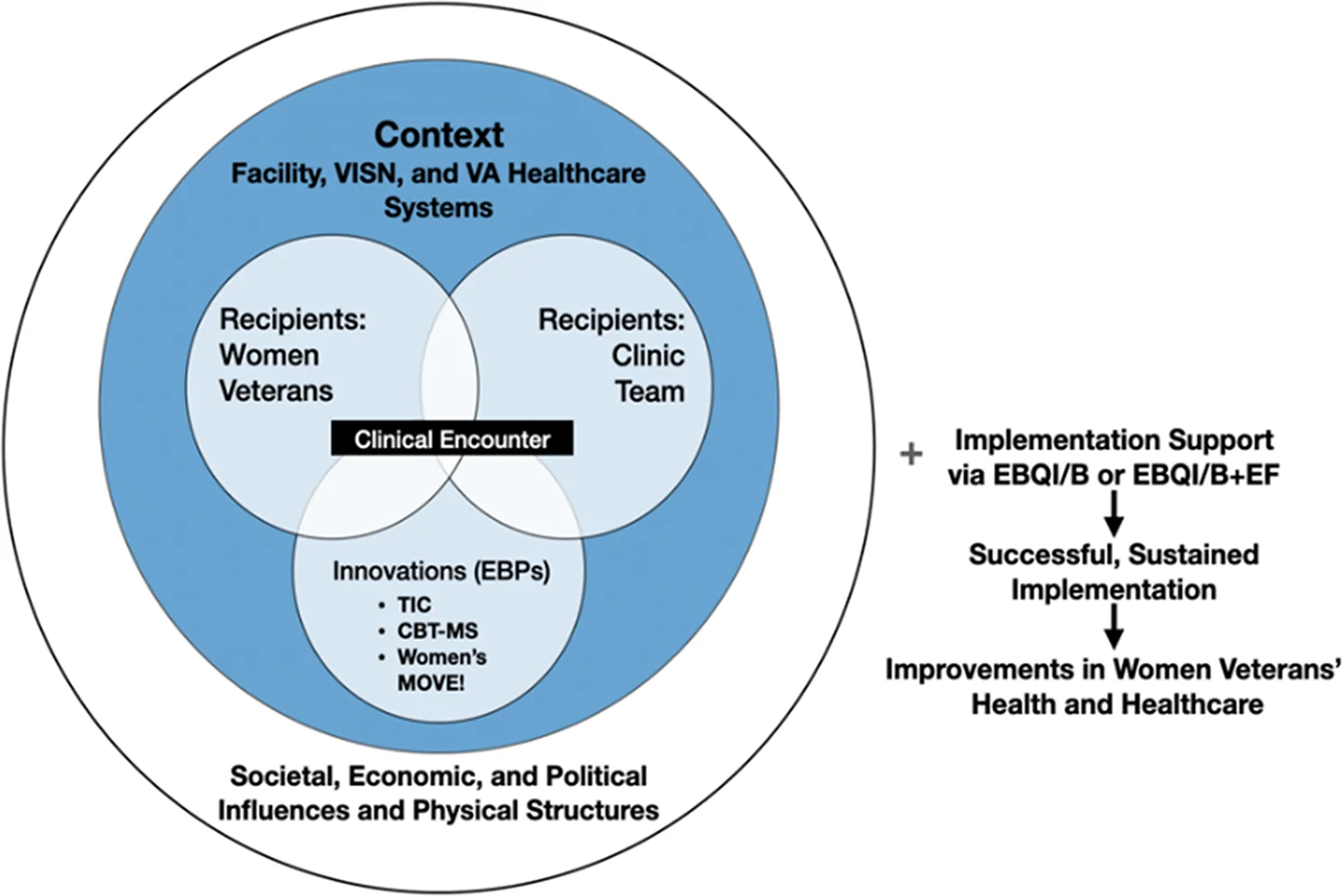
EMPOWER 3.0 Conceptual Model

*EMPOWER 3.0 Conceptual Framework*. Our conceptual model is grounded in the Health Equity Implementation Framework (HEIF, Fig. 3) [52], which integrates implementation determinants common across implementation theories, models, and frameworks, such as context, recipients, and characteristics of the innovation, and augments them with consideration of how these features intersect in the unique context of the clinical encounter.

*Aim 2a. Improved access to care that is trauma-informed and tailored for women Veterans: Data sources*. We will use the VA CDW platform to collect EHR data from CPRS/VistA (VA legacy EHR) and FEHR (new VA Federated EHR) to measure patient access and engagement in care. Based on input from the Learning Community, we will monitor changes and trends in VA performance metrics including measures from Veteran-Signals (V-Signals, such as Well-Being Signs); the Reporting, Analytics, Performance, Improvement & Deployment’s Electronic Quality Measurement (eQM) portal; the External Peer Review Program (EPRP); and the Survey of Patient Healthcare Experiences (SHEP). We will use PGHD [46] to collect weight measurements from the wireless scales for Women’s MOVE! participants. For each EBP, we will collect PROs from brief patient surveys administered on REDCap [45]. Site-level measures will come from VA administrative and OWH data.

*Aim 2a. Measures*. Measures across EBPs and their data sources are shown in Table 2. The *primary outcome* of interest is engagement in the EBPs. Because collaboration and empowerment in the clinical encounter are central principles of TIC [53] and shared decision-making is a core component of successful TIC implementation [54], TIC engagement is defined as the number of female patients with encounters that include shared decision-making as indicated by the completion of the Well-Being Signs (WBS) measure, a validated three-item measure of patients’ psychosocial well-being used in treatment planning. This national measure was designed and validated to provide insight into patients’ psychosocial well-being in terms of health, vocation, finances, and social relationships to aid in shared decision-making to ensure that treatment plans are optimized [55]. For CBT-SM and Women’s MOVE!, the *primary outcomes* are EBP engagement defined as participation in one or more sessions. MOVE! session participation is documented with primary stop codes (372 and 373) and encounter coding for CBT-SM will be developed with OMH partner guidance. We will create composite measures of the proportion of women participating in the EBPs (#participate/#eligible): all women in midlife (age 45–65 with the inclusion of women of any age experiencing early or surgical menopause for CBT-SM) with at least one primary care or PCMHI visit over the implementation period will be eligible. We will also collect additional *participation and engagement* measures for each EBP. For TIC, measures include care team participation in training, TIC knowledge and confidence ratings, and number of initiatives completed in three other TIC components (staff education, self-care activities for staff, and safe patient care environment). For CBT-SM and Women’s MOVE!, measures include average number of sessions completed. Because the impact goal is to improve experience of the clinical encounter, we will collect *PROs* including safety, trust, and satisfaction reports after appointments for TIC, change in physical and clinical symptoms for CBT-SM, and change in physical activity and weight for Women’s MOVE!. Because availability of new EBPs often impact health service *utilization*, we will examine no-show rates for appointments and attrition from VA rates [56] (for TIC), participation in menopause-related services (for CBT-SM; e.g., Whole Health, primary care, gynecology care, HT use), and medication and other weight management or health behavior/physical activity programs (for Women’s MOVE!) [18].

Secondary outcomes of interest also include *clinical outcomes/performance measures*. As a primary impact goal is to improve women Veterans’ experience of the clinical encounter, we will measure sense of well-being (WBS score) and trust (e.g., overall trust, ease/access, effectiveness/satisfaction, and emotion measures in V-Signals) [57]. Examples of clinical outcomes include prevalence of hypertension, pre-diabetes and diabetes, and incident diabetes based on diagnosis codes, medications, and laboratory records [18], documentation of HT and bone density screening, and VA performance measures such as preventive screening (e.g., breast cancer) and Healthcare Effectiveness Data and Information Set (HEDIS) performance measures for diabetes and hypertension. We will include *other measures* at the site, provider, and patient levels available from VA administrative data and our operations partners. For the sites, we will include facility type (VAMC or CBOC) and size (# of unique WV patients), year of WV-ACHIEVE program participation, women’s health primary care program model (mixed-sex primary care [Model 1] vs. some women-specific care [Model 2] vs. comprehensive women’s health clinic [Model 3]), number of designated women’s health providers, and rurality. Provider characteristics include provider type and whether the provider is a designated women’s health provider. Patient-level controls will include sociodemographics (e.g., age, race/ethnicity), comorbidities (e.g., diabetes, cardiovascular risk), service-connected disability status, healthcare utilization, and menopause-related ICD-10 (e.g., N95, E28). We will include *other measures* at the site, provider, and patient levels from VA administrative data and our operations partners.

**Table 2:** Measures for Aim 2a: Improved Access to Care That Is Trauma-Informed and Tailored for Women Veterans

	TIC	CBT-SM	Women’s MOVE!	Data Sources
Primary Outcome: Engagement	• # of women with shared decision-making treatment planning marker (1 = WBS completed in VA EHR)	• # women participating in CBT-SM (yes = one or more sessions)	• # of women participating in Women’s MOVE! (yes = one or more sessions)	• VA EHR
Participation and Engagement	• # care team trained • TIC knowledge and confidence • # initiatives completed within and across TIC components	• Average # of sessions completed	• Average # of sessions completed	• TIC/CBT-SM/Women’s MOVE! data collection • VA EHR
Patient- Reported Outcomes	• Safety, trust, and satisfaction reports after appointments	• Change in reported physical and clinical symptoms (e.g., bothersome)	• Change in reported physical activity • % weight change at 6- & 12- months	• Patient surveys/ Patient Generated Health Data
Utilization	• Reduction in no-show rates • Reduction in attrition rates	• Participation in menopause-related services (e.g., Whole Health, primary care/gyn care) • Hormone therapy use	• Increased participation in weight management services/ programs (e.g., nutrition/ dietetics, medications)	• VA EHR
Clinical Outcomes/ Performance Measures	• WBS Scores • Trust, satisfaction, ratings of providers • Quality: care coordination, continuity of care, communication • Preventive screenings and follow-up (e.g., breast, cervical, colorectal cancer)	• WBS Scores • CVD risk: overweight/ obesity, hypertension • Bone density screening (60+) • Preventive screenings • Hypertension and diabetes treatment (HEDIS) • Whole Health participation	• WBS Scores • CVD risk: overweight/ obesity • Prediabetes, diabetes care and control • Hypertension and diabetes treatment (HEDIS) • Whole Health or NCP/other health behavior participation	• VA EHR Veteran-Signals • Performance measures from the Office of Quality and Patient Safety
Other measures	• Site: facility type (VAMC or CBOC), size (# of unique women Veteran patients), year in WV-ACHIEVE, women’s health primary care program model (mixed-sex primary care, some women-specific care, comprehensive women’s health clinic), rurality • Provider: provider type, designated women’s health provider • Patient: sociodemographics (e.g., age, race/ethnicity), comorbidities (e.g., diabetes, cardiovascular risk), service-connected disability status, healthcare utilization, and menopause related ICD-10			

CBT-SM: Cognitive Behavioral Therapy for Symptoms of Menopause; CVD: Cardiovascular Disease; EHR: Electronic Health Record; HEDIS: Healthcare Effectiveness Data and Information Set; ICD-10: International Classification of Diseases 10th revision; MOVE!: Managing Overweight and Obesity for Veterans Everywhere; NCP: National Center for Health Promotion and Disease Prevention; TIC: Trauma Informed Care; WBS: Well Being Signs; WV-ACHIEVE: Women Veterans Achieving Comprehensive Health Innovation and Enhancement for Women Veterans through EBQI

*Data Analysis*. We will use generalized linear models to evaluate the effectiveness of implementation strategies on EBP engagement. The models will account for clustering at the site level as well as incorporating site-, provider-, and patient-level covariates. The main independent variable of interest is implementation using EBQI/B versus EBQI/B + EF, and the primary outcomes will include the composite measure of the engagement in the EBPs (#participate/ #eligible; see above). In each model, we will examine the potential moderating effects of site-level characteristics (e.g., facility type, size, women’s health care program model, etc.). Incorporation of the clustering in the model as well as the estimation of the model parameters will be performed using Stata v19 (Stata Corp, College Station, TX). We will evaluate goodness-of-fit using Mallows statistic (Cp). We will conduct subgroup analyses to examine if effects of EBQI/B versus EBQI/B + EF vary by EBP and evaluate effects on *utilization and clinical outcomes/performance measures*.

*Aim 2a. power calculations*. We based the power calculations on the study’s basic single level of clustering sample design, where 18 sites are randomized to receive either EBQI/B or EBQI/B + EF to implement the EBPs. To detect a 0.14 difference in the proportion of women who participate between EBQI/B or EBQI/B + EF, with cluster adjustment (ICC=0.01 based on prior work [33]), we will reach 90% power by recruiting 40 women per site (total of 720 women). Even though two sites (CBOCs) will be randomized along with the VAMC with the same leadership, all 18 sites are considered separate clusters for these calculations.

*Aim 2b. implementation strategy fidelity, speed of implementation, and sustainment*. The Stages of Implementation Completion^®^ (SIC) [58] is an assessment tool developed to evaluate progress and milestones in implementing EBPs across diverse settings and strategies. The SIC spans eight standardized stages (e.g., engagement), populated by activities that are largely universal in implementation efforts, and measures the percentage of activities completed, time (days) to completion, and site fidelity to implementation strategies, enabling comparison of sites’ progression to competence (i.e., ability to routinely deliver the EBP) and sustainment. In EMPOWER 2.0, we partnered with SIC developer Dr. Lisa Saldana to adapt the tool for VA use, accounting for multiple levels of leadership and varied organizational structures [59]. For EMPOWER 3.0, we will work with Dr. Saldana to update the EMPOWER-SIC to assess EBQI/B and EBQI/B + EF strategies; our implementation teams are experienced in use of the SIC and will gather the information necessary to populate the assessment tool for each site via Aim 2c data collection. Sustainment will be defined by sites’ reported intention to sustain (yes/no) and continued monitoring of EBP primary outcomes (see Table 2).

*Aim 2b Analysis*: SIC allows for calculation of each site’s: *stage score*, which describes the ultimate stage achieved at a given site; *fidelity score*, which describes the proportion of activities completed by a site within a given stage/phase; and *duration score*, which describes the amount of time a site spends in each stage.

*Aim 2c. Recipient perspectives on and experiences of EBP implementation*: How innovation recipients perceive EBPs and experience their implementation is critical to successful implementation and sustainment [60]. The HEIF conceptualizes both clinical teams implementing and patients receiving an intervention as “recipients,” while also acknowledging the critical role of individuals with social or structural influence on context (e.g. facility- or VISN-level leadership) [52]. We will therefore assess perspectives on and experiences of implementation through interviews and brief structured data collection with innovation recipients and multilevel VA leadership, i.e.: (1) *Women Veterans* receiving VA care at participating sites; and (2) *Clinical Teams* (e.g., WH-PBRN Site Leads, primary care providers, PCMHI care providers, MOVE! coordinators) and *Multilevel Leadership* (e.g., VISN and facility leaders).

*Women Veterans* will participate in semi-structured interviews at two timepoints. In Year 1, during co-production, 20 midlife women VA users (five per VISN) for each EBP (*n* = 60 total) will be asked to describe their experiences of relevant VA care (i.e., primary care, menopause, and weight management), provide feedback on planned EBPs, and offer recommendations for ensuring each EBP is trauma-sensitive and inclusive. We will identify women Veteran VA users in our sites with markers for EBP eligibility. Post-implementation, we will conduct semi-structured interviews with a subsample of women (20 per EBP, *n* = 60 total) to assess satisfaction and acceptability. We will purposively select among a random sample of EBP participants across sites. CBT and Women’s MOVE! participants will be stratified by completion of one session vs. completion of four sessions (for CBT) and eight sessions (for Women’s MOVE!) to explore factors influencing completion. Interviews will cover EBP experiences, attitudes, perceived impacts, barriers and facilitators to completion, and recommendations for future improvements. To ensure diverse perspectives, recruitment at both timepoints will seek maximum variation in racial/ethnic background and urban/rural residence. Interviews will be conducted virtually using MS Teams and last ~ 45 min; participants will be compensated $50.

Individuals in *Clinical Teams* (e.g., PACT teamlet/extended team) and *MultilevelLeadership* (e.g., Medical Center Director, VISN WVPM) will also be invited to participate in semi-structured interviews at two timepoints. Participants will be identified using publicly available information, lists provided by site-identified QI champions, and snowball sampling, whereby participants will be asked to recommend others with relevant perspectives. We estimate approximately six *clinical team* and *leadership* participants per site (*n* = 108) and five per VISN (*n* = 20) for a total sample at pre-implementation of *n* = 128. A similar sample size is anticipated for post-implementation interviews; this sampling approach ensures high information power (i.e., participants with the most relevant expertise/influence) and analytic data sufficiency [13, 61, 62].

*Pre-implementation interviews* will explore usual care at baseline for the relevant condition, HEIF domains (e.g., context, perceived characteristics of innovations/EBPs), and characteristics of recipient patients (e.g., rural/urban) and providers (e.g., prior training) using semi-structured interview guides informed by our prior work [12, 13]. *Post-implementation interviews* will assess HEIF domains and recommendations for EBP adaptations and/or spread. In both pre- and post-implementation interviews, we will also inquire about factors relevant to the business case for each EBP, such as associated costs and benefits, time/effort involved in initial implementation vs. sustainment, and financial/ non-financial benefits (e.g., performance metrics, patient outcomes.) These data will inform cost analysis [63] allowing us to tailor the T2P-informed scale-up plan and business case for each EBP by providing information for diverse audiences and partners in VA.

*Periodic reflections and structured note templates*. In addition to semi-structured interviews, our EMPOWER team has pioneered use of pragmatic, low-burden, ethnographic methods to document implementation events, challenges, opportunities, and experiences throughout implementation [64]. *Periodic reflections* have been widely adopted in implementation studies [65, 66] and shown to support timely and accurate documentation of dynamic implementation activities, strategies, adaptations, and changes to context. Periodic reflections will be completed with *EMPOWER PIs and Co-PIs* and *members of the IDC Implementation Support Team* for each EBP and implementation strategy. Conducted monthly via telephone/MS Teams, reflections will follow a standardized template and last 15–60 min, depending on the amount of current activity and number of participants (range 1–4). We have also developed *structured note templates* (“call sheets”) [67] to ensure fidelity to implementation strategy (“nudging” key activities per time point) while documenting site contacts and meetings [50, 67]. Adapted for EBQI/B and EF activities, call sheets will be used in QI Collaborative and EF calls to track attendance, site updates, and support requests, as well as open-ended, templated notes on implementation progress at each site. Call sheets will be completed by QI Collaborative/EF Leads and used to record SIC progression (e.g., key dates), dose and intensity of contacts, and qualitative observations, supporting cost estimates for each strategy, EBP, and site. We will also document dialogue and feedback using a structured note template in EMPOWER 3.0 Learning Community meetings.

*Aim 2c analysis plan*: All *semi-structured interviews* will be digitally recorded, transcribed verbatim by an approved transcriptionist, and reviewed for accuracy. Following recently published recommendations for rigor [68], we will use rapid qualitative analysis to generate timely results and inform implementation processes. For example, during **co-production**, we will analyze women Veteran interviews and Learning Community feedback to inform EBP tailoring; we will also analyze periodic reflections with EBP PIs and other implementation team members during this period to understand how feedback was integrated [65]. In **pre-implementation**, we will create *Rapid Implementation Feedback (RIF) reports* [69] to integrate key contextual information, such as workflow and critical roles, for site-specific planning. These reports synthesize findings, prioritize topics, and provide up-to-date summaries of site readiness and preferences. **Post-implementation** (and in other instances in which in-depth analysis is required, e.g., to better understand unexpected challenges), we will conduct in-depth content analysis using ATLAS.ti [70]. A codebook based on HEIF constructs and interview guides will evolve with emergent themes. Data will be compared across sites (e.g., urban vs. rural), strategies, and time, integrating *periodic reflections* and *structured notes* to assess implementation experiences, adaptations, and change [15, 71].

*Aim 2d. Costs of implementation and business case analysis to support sustained EBP use* We will achieve Aim 2d, comparing EBQI/B and EBQI/B + EF costs, in two steps: (1) conducting *cost analyses* using the *Cost of Implementing New Strategies* (COINS) measure, a SIC add-on that tracks participant time investment at sites and within the EMPOWER team [72]; and (2) integrating implementation costs with data on costs and benefits of EBPs (TIC, CBT-SM, MOVE!) to create a *business case analysis* for six strategy-EBP combinations (e.g., EBQI/B + TIC). COINS, supplemented by data from interviews and reflections related to implementation costs, will provide cost estimates for each implementation phase. These cost estimates will inform scale-up plans and timelines for future adopters to be developed in Aim 3. Scale-up plans will detail costs, personnel effort (e.g., percent full-time equivalent), and business cases for EBP implementation. Business cases will analyze upfront implementation costs, ongoing delivery costs, and downstream impacts (e.g., for Women’s MOVE!, virtual session delivery costs vs. diabetes-related healthcare reductions). Non-financial benefits relevant to operational partners, such as improved program participation rates and performance measure scores, will also be considered. The business case will also include budget impact analyses (BIA), comparing usual care costs with EBP implementation and delivery costs to assess short-term effects on healthcare resource use [73].

*Aim 2d Measures*: Requirements for BIA include estimates of eligible population, current treatment mix and associated costs, expected treatment mix post-implementation and associated costs, and estimated changes in condition-related downstream costs [73]. Cost estimates for each strategy will be informed by COINS data (e.g., implementation dose in #hours/per site). Healthcare utilization data will be drawn from CDW and integrated with COINS implementation cost estimates for each strategy-EBP combination.

*Aim 2d Analysis*: Analyses will examine the comparative cost and outcomes of EBQI/B and EBQI/B + EF for each EBP. Qualitative data will shed light on the “qualitative residual” that may be underexplored in traditional economic evaluations, resulting in improved cost estimates [63].

### Aim 3. In collaboration with women veterans and site-, VISN- and national partners, develop a Translation-to-Policy (T2P)-informed scale-up and evaluation plan [27]

Drawing on lessons from EMPOWER 2.0’s implementation across 22 sites and prior collaborative projects with OWH and OMH, we aim to leverage partnerships and the Learning Health System model [74] to support sustainment, spread, and continuous improvement of trauma-informed EBPs in VA care.

In EMPOWER 3.0, we will formalize scaling efforts using the *T2P Learning Cycle* framework [27]. This framework delineates three iterative phases. In *Performance to Data*, a Learning Community identifies datasets to evaluate quality and outcomes gaps, co-designing implementation and policy goals aligned with system goals and priorities. In *Data to Knowledge*, data integration and external evidence review guide testing of novel and/or implementing existing EBPs. Our team has already launched these two phases, developing the EMPOWER 3.0 Learning Community and engaging with partners to conduct needs and assets assessment and identify EBPs for implementation. Thus, the proposed hybrid type 3 effectiveness-implementation trial represents the *Knowledge to Performance* phase, launching EBP implementation and QI initiatives consistent with our EBQI-based implementation strategies. In Aim 3, we will work directly with our Learning Community and additional partners as needed to co-design scale-up and evaluation plans, including evaluation metrics, data sources, and dashboards for monitoring and addressing gaps or disparities. These efforts in turn will inform the next *Data to Knowledge* phase, supporting QUERI’s goal of improving Veteran health “by more rapidly translating evidence-based innovations into routine practice” [27].

## Discussion

Relatively few studies have compared the effectiveness of implementation strategies for achieving rollout of multiple evidence-based interventions across large healthcare systems. This study protocol provides an innovative model for conducting partnered and collaborative co-production to enhance the acceptability, effectiveness, and sustainability of EBP packaging and workflows as well as multi-method evaluation in a national healthcare network.

Although this study is conducted entirely within the VA healthcare system and findings may not fully generalize to other settings, considerable site- and regional-level variation within the VA using 18 sites from four VISNs is expected to yield insights applicable to common challenges in responding to local needs and resources. This study leverages a unique opportunity to expand upon VA OWH’s large-scale investment in building capacity for EBQI across VA women’s health, and to assess what additional supports are needed to support previously EBQI-trained sites in implementing and sustaining novel interventions. Conducting rigorous comparison of clinical benefit, cost and return on investment, and time to achieving implementation associated with more and less intensive versions of supplemental EBQI (with and without external facilitation) will directly support evidence-based policy and resource allocation decisions. We also note that this is among the first multi-intervention implementation trials to center patients’ trust, well-being, and engagement in care as primary outcomes, directly examining the impact of implementation efforts on these critical drivers for long-term patient health.

## Supplementary Information

Below is the link to the electronic supplementary material.

Supplementary Material 1

## Data Availability

No datasets were generated or analysed during the current study.
