# Supplementary material for: Enhancing mental and physical health of women through engagement and retention (EMPOWER) 3.0 QUERI: study protocol for a cluster-randomized hybrid type 3 implementation-effectiveness trial
Source: Implement Sci. 2026 Jul 8;21:55. doi: 10.1186/s13012-026-01521-3 (PMC13632245; doi:10.1186/s13012-026-01521-3)
Supplement: Supplementary file 1 [file 13012_2026_1521_MOESM1_ESM.docx]

**Additional file 1 – EMPOWER 3.0 Evidence-Based Practices (EBPs) and Related Health Conditions**

Women Veterans are the fastest growing group of Veterans Health Administration (VA) users, with most users in midlife. More women Veterans than ever are now using VA, and the proportion of VA users who are women has also increased, from 5.9% in FY10 to 9.2% in FY20 [1]. In FY20, 41% of women Veteran VHA users were 18-44 years old, 45% were 45-64 years old, and 15% were 65+ years old. From FY10 to FY20, the number of women in midlife increased 1.8-fold. The proportion of women Veteran VA users in midlife who were Veterans of color also increased in that decade, from 36% to 43%.

Few VA improvement efforts have focused on women Veterans’ health and healthcare in midlife. Despite the preponderance of women Veteran VA users in midlife, a recent VA evidence synthesis revealed that few studies or quality improvement (QI) initiatives have focused on middle-aged women’s health conditions and healthcare utilization and experience [2]. During midlife, “health issues can become more prominent, including menopause and the need for preventive care measures to avert late-life disease” [1]. In midlife, women Veterans and non-Veterans have the highest obesity rates, 75% of women have at least one major cardiovascular risk (e.g., hypertension), and the prevalence of diabetes rises to 16%, compared to 11% among Veterans overall [3,4]. Women’s health in midlife is getting national attention: a 2024 White House Executive Order [5] called for evidence-based practices (EBPs) “to improve women’s experiences in the menopausal and perimenopausal periods.” The proposed EMPOWER 3.0 QUERI responds directly to escalating calls for efforts to mitigate critical gaps in care for women Veterans in midlife.

Women Veterans’ engagement and retention in care—throughout the adult life span—are of primary importance and relate strongly to trust. We conceptualize engagement as “patients, families, their representatives, and health professionals working in active partnership…to improve health and health care” [6]. Trust, central to women Veterans’ engagement, grows when providers offer effective communication, care tailored for women’s needs, and trauma-informed interactions [7]. Conversely, distrust—linked to discrimination, harassment, and trauma (e.g., military sexual trauma [MST])—leads to delayed or missed care and reduced well-being [8,9]. Research indicates that the strongest negative impact of trauma during military service on health status is found among women aged 45-64 [10]. Women Veterans who experienced institutional betrayal related to MST disclosures while still in the military report lower willingness to use VA care and higher willingness to use non-VA care [11], and a subset report reservations about using VA care related to concerns about distrust, provider compassion, privacy, stigma, shame, and continuity of care [12]. As more women Veterans than ever are using VA for their care, it is incumbent to ensure that Veterans’ trust is fostered and maintained in healthcare informed by the pervasiveness of trauma [13].

This EMPOWER 3.0 proposal addresses women Veterans’ healthcare needs in midlife, emphasizing engagement and trust as VA priorities. In planning for this proposal, our leadership team built upon prior work in EMPOWER 1.0 and 2.0 [14–22], and collaborated with EMPOWER 2.0 operations partners and the VA Women’s Improvement Network (WIN, a national group of women Veteran VA users) to identify implementation opportunities and assess assets and needs. The focus on midlife received broad support, highlighting interests in menopause and weight management as well as the impacts of trauma on virtually all health conditions faced by women. To address these needs and support chronic disease prevention in midlife and beyond, we propose: (1) increasing VA capacity to deliver trauma-informed primary care (TIC) for women Veterans; (2) enhancing care options during the menopause transition; and (3) tailoring VA’s existing MOVE! program to better meet women Veterans’ needs in weight management. Below we describe the evidence and partner priorities related to these three areas, followed by a description of each EBP.

Trauma-informed care (TIC) provides a valuable set of principles for improving trust, well-being, and engagement in VA care. Trauma, experienced by upwards of two thirds of women Veterans [10], can be conceptualized as event(s) “experienced by an individual as physically or emotional harmful or life threatening” that have “lasting adverse effects on the individual’s functioning and mental, physical, social, emotional, or spiritual well-being” [23]. TIC refers to a broad systems approach that “integrates trauma-informed practices (acknowledging that potentially traumatic exposures have taken place) throughout a service delivery system” [24]. While TIC implementation may differ by setting (e.g., primary care, emergency medicine) and population (e.g., youth, Veteran), multiple systematic reviews have found that integrating trauma-informed principles into healthcare delivery can improve care experiences of both patients and providers and result in improved healthcare delivery and health outcomes for patients [25–27]. It can also reduce re-traumatization experiences within healthcare settings by removing or reducing potential trauma-related triggers in the environment [28]. Six key principles characterize TIC: (1) safety, (2) trustworthiness and transparency, (3) peer support, (4) collaboration and mutuality, (5) empowerment and choice, and (6) acknowledgement of cultural and historical issues [23].

In 2021, VHA adopted TIC as an overarching care model, mandating that “clinicians should proactively adopt a trauma-informed approach with all Veterans,” with a focus on “establishing a safe environment, conveying trustworthiness, and promoting choice, collaboration, and empowerment” [29]. TIC has particular value in primary care, where providers face the challenge of accurately identifying and negotiating effective treatment plans for health problems that may be rooted in or amplified by past trauma exposure, including obesity, diabetes, depression, substance use, and cardiovascular disease [28,30]. Furthermore, hands-on physical exams, routine in primary care, may be retraumatizing if approached in a non-trauma-informed manner. Although VA offers a variety of resources to support TIC approaches, VA medical centers, like other healthcare settings [31], continue to report a variety of barriers to widespread TIC implementation. A practice scan conducted by the Women’s Health Practice-Based Research Network (WH-PBRN, comprising 76 VA sites) found that more than 90% of responding sites (86% response rate) reported need for ongoing TIC training opportunities, support for improving environments of care, and concrete guidance (e.g., checklists) for implementing and sustaining TIC [32]. Implementation of TIC in EMPOWER 3.0 could serve as a foundation for implementation efforts throughout the learning health system.

Women Veterans need enhanced care options during the menopausal transition. The menopausal transition is a key phase in the reproductive life cycle of biologically female individuals, marked by hormonal changes and symptoms that may include vasomotor (VMS, e.g. “hot flashes,” “night sweats”), vaginal, sleep, and emotional disruptions [33]. While hormone therapy (HT) remains the most effective intervention for VMS and vaginal symptoms, and may aid in preventing chronic conditions [34], comprehensive care programs are needed, particularly since some women may not be candidates for HT based on risk factors and/or personal preference [35]. Although women Veterans receive HT at rates comparable to or higher than the non-Veteran population [36], women in and out of the VA seek evidence-based non-hormonal treatment options [37,38]. In a recent WH-PBRN practice scan, VA women’s health (WH) providers identified a need for additional care resources for menopause symptoms, specifically including “menopause-specific training, educational materials for Veterans, and clinical resources to treat common menopause symptoms including sexual function, difficulty sleeping, mood swings, urinary incontinence, and hot flashes” [39]. Furthermore, HT is associated with increased risk of suicide attempt and death by suicide among women Veterans [40], suggesting that mental health interventions may be needed in this period. These findings underscore the importance of access to evidence-based mental health care during the menopausal transition for women Veterans.

Women Veterans in midlife need VA-based weight management and well-being support. Obesity affects 49% of midlife women Veterans in VA care [41], and is linked to trauma, chronic conditions (e.g. diabetes, heart disease, posttraumatic stress disorder (PTSD), pain), and reduced quality of life [42,43]. Although VA’s MOVE! (Managing Overweight and Obesity for Veterans Everywhere) program is effective for weight management, tailoring is needed to better serve women Veterans. Evaluations of MOVE! indicate that, while both men and women lose weight [44,45], women lose less weight and attend fewer sessions than men [44], and report challenges like emotional eating, loneliness, low self-confidence [44], and discomfort with mixed-gender classes that can trigger PTSD symptoms [46]. Weight discrimination during and after military service [47], including in VA [8], may further erode trust and willingness to engage in weight management programs. We demonstrated in EMPOWER 1.0 and 2.0 that tailored weight management support was preferred by women Veterans and led to increased engagement in healthy lifestyle changes [14,18].

**References**

1. Breland J, Frayne S, Saechao F, Gujral K, Vashi A, Shaw J, et al. Sourcebook: Women Veterans in the Veterans Health Administration. Volume 5: Longitudinal Trends in Sociodemopgrahics and Utilization, Including Type, Modality, and Source of Care. Washington, D.C.: Women’s Health Evaluation Initiative, Office of Women’s Health, Veterans Health Administration, Department of Veterans Affairs.; 2024 June.

2. Pace R, Dancu C, Raman S, et al. An Evidence Map of the Women Veterans’ Health Literature (2016-2023). Washington, D.C.: Evidence Synthesis Program, Health Services Research, Office and Research and Development, Department of Veterans Affairs.; 2024. Report No.: VA ESP Project #09-010.

3. Han JK, Yano EM, Watson KE, Ebrahimi R. Cardiovascular Care in Women Veterans: A Call to Action. Circulation. 2019;139:1102–9. https://doi.org/10.1161/CIRCULATIONAHA.118.037748

4. Emmerich S, Fryar C, Stierman B, Ogden C. Obesity and Severe Obesity Prevalence in Adults: United States, August 2021–August 2023 [Internet]. National Center for Health Statistics (U.S.); 2024 Sept. https://doi.org/10.15620/cdc/159281

5. Executive Order on Advancing Women’s Health Research and Innovation [Internet]. 2024 [cited 2024 Oct 24]. https://www.whitehouse.gov/briefing-room/presidential-actions/2024/03/18/executive-order-on-advancing-womens-health-research-and-innovation/. Accessed 24 Oct 2024

6. Carman KL, Dardess P, Maurer M, Sofaer S, Adams K, Bechtel C, et al. Patient And Family Engagement: A Framework For Understanding The Elements And Developing Interventions And Policies. Health Affairs. 2013;32:223–31. https://doi.org/10.1377/hlthaff.2012.1133

7. Carlson GC, Than CT, Rose D, Brunner J, Chanfreau-Coffinier C, Canelo IA, et al. What Drives Women Veterans’ Trust in VA Healthcare Providers? Womens Health Issues. 2022;32:499–508. https://doi.org/10.1016/j.whi.2022.02.004

8. Katon JG, Benson SK, Sriskantharajah V, Callegari LS, Fenwick K, Gray KE, et al. Experiences of Discrimination Among Women and Gender Diverse Veterans Using Veterans Health Administration Health Care. Health Equity. 2024;8:692–700. https://doi.org/10.1089/heq.2024.0085

9. MacDonald S, Judge-Golden C, Borrero S, Zhao X, Mor MK, Hausmann LRM. Experiences of Perceived Gender-based Discrimination Among Women Veterans: Data From the ECUUN Study. Medical Care. 2020;58:483–90. https://doi.org/10.1097/MLR.0000000000001304

10. Gibson CJ, Gray KE, Katon JG, Simpson TL, Lehavot K. Sexual Assault, Sexual Harassment, and Physical Victimization during Military Service across Age Cohorts of Women Veterans. Women’s Health Issues. 2016;26:225–31. https://doi.org/10.1016/j.whi.2015.09.013

11. Monteith LL, Holliday R, Schneider AL, Miller CN, Bahraini NH, Forster JE. Institutional betrayal and help-seeking among women survivors of military sexual trauma. Psychological Trauma: Theory, Research, Practice, and Policy. 2021;13:814–23. https://doi.org/10.1037/tra0001027

12. Monteith LL, Bahraini NH, Gerber HR, Dorsey Holliman B, Schneider AL, Holliday R, et al. Military sexual trauma survivors’ perceptions of Veterans Health Administration care: A qualitative examination. Psychological Services. 2020;17:178–86. https://doi.org/10.1037/ser0000290

13. Department of Veterans Affairs Fiscal Years 2022-2028 Strategic Plan [Internet]. Washington, D.C.: Department of Veterans Affairs; https://department.va.gov/wp-content/uploads/2022/09/va-strategic-plan-2022-2028.pdf. Accessed 2 Dec 2024

14. Dyer KE, Moreau JL, Finley E, Bean-Mayberry B, Farmer MM, Bernet D, et al. Tailoring an evidence-based lifestyle intervention to meet the needs of women Veterans with prediabetes. Women & Health. 2020;60:748–62. https://doi.org/10.1080/03630242.2019.1710892

15. Brunner J, Farmer MM, Bean-Mayberry B, Chanfreau-Coffinier C, Than CT, Hamilton AB, et al. Implementing clinical decision support for reducing women Veterans’ cardiovascular risk in VA: A mixed-method, longitudinal study of context, adaptation, and uptake. Front Health Serv. 2022;2:946802. https://doi.org/10.3389/frhs.2022.946802

16. Than CT, Bean-Mayberry B, Schweizer CA, Lee M, Chanfreau-Coffinier C, Clair K, et al. Ask and Ask Again: Repeated Screening for Smoking Increases Likelihood of Prescription for Cessation Treatment Among Women Veterans. J Gen Intern Med. 2023;38:2553–9. https://doi.org/10.1007/s11606-023-08227-y

17. Damschroder LJ, Hamilton A, Farmer MM, Bean-Mayberry B, Richardson C, Chanfreau C, et al. Real-world impacts from a decade of Quality Enhancement Research Initiative-partnered projects to translate the Diabetes Prevention Program in the Veterans Health Administration. Health Serv Res. 2024;59 Suppl 2:e14349. https://doi.org/10.1111/1475-6773.14349

18. Malone A, Clair K, Chanfreau C, Bean-Mayberry B, Oberman R, Lesser R, et al. Predictors of enrollment in a virtual diabetes prevention program among women veterans: a retrospective analysis. BMC Women’s Health. 2024;24:465. https://doi.org/10.1186/s12905-024-03314-6

19. Finley EP, Hamilton AB, Canelo I, Jackson LSS, Lesser R, Oberman RS, et al. Collaborative tailoring of the Reach Out, Stay Strong Essentials (ROSE) program for pregnant veterans in the U.S. Veterans Health Administration: a qualitative case study of contextual conditions and adaptations. BMC Health Serv Res. 2025;25:746. https://doi.org/10.1186/s12913-025-12871-x

20. Clair K, Fletcher E, Oishi A, Schweizer C, Jackson L, Chanfreau C, et al. “It’s Just a Matter of Doing It”: Women Veterans’ Experiences with Changing Behavior for Cardiovascular Risk Reduction. J Gen Intern Med. 2026 Mar.

21. Clair K, Bean-Mayberry B, Chanfreau C, Hurley S, Pear E, Goldstein MG, et al. Reaching Veterans Most in Need: Evaluating Engagement in a National Telephone Lifestyle Coaching Program for Veterans. J Gen Intern Med [Internet]. 2026 [cited 2026 Mar 26]; https://doi.org/10.1007/s11606-026-10299-5

22. Farmer MM, Hamilton AB, Finley EP, Lee ML, Chanfreau C, Than C, et al. Implementation of a cardiovascular toolkit in primary care increased women Veterans’ engagement in behavior change programs: results from a non-randomized stepped wedge trial. BMC Prim Care. 2026;27:95. https://doi.org/10.1186/s12875-025-03158-5

23. Substance Abuse and Mental Health Services Administration. SAMHSA’s Concept of Trauma and Guidance for a Trauma-Informed Approach. [Internet]. Rockville, MD: Substance Abuse and Mental Health Services Administration; 2014. Report No.: HHS Publication No. (SMA) 14-4884. https://store.samhsa.gov/sites/default/files/sma14-4884.pdf. Accessed 2 Nov 2024

24. National Strategy for Trauma-Informed Care Operating Plan [Internet]. Substance Abuse and Mental Health Services Administration (SAMHSA),; 2022. https://www.samhsa.gov/sites/default/files/trauma-informed-care-operating-plan.pdf. Accessed 2 Nov 2024

25. Mahon D. An Umbrella Review of Systematic Reviews on Trauma Informed Approaches. Community Ment Health J [Internet]. 2024 [cited 2024 Nov 2]; https://doi.org/10.1007/s10597-024-01317-z

26. Goldstein E, Chokshi B, Melendez-Torres G, Rios A, Jelley M, Lewis-O’Connor A. Effectiveness of Trauma-Informed Care Implementation in Health Care Settings: Systematic Review of Reviews and Realist Synthesis. TPJ. 2024;28:135–50. https://doi.org/10.7812/TPP/23.127

27. Fernández V, Gausereide-Corral M, Valiente C, Sánchez-Iglesias I. Effectiveness of trauma-informed care interventions at the organizational level: A systematic review. Psychological Services. 2023;20:849–62. https://doi.org/10.1037/ser0000737

28. Machtinger EL, Cuca YP, Khanna N, Rose CD, Kimberg LS. From Treatment to Healing: The Promise of Trauma-Informed Primary Care. Women’s Health Issues. 2015;25:193–7. https://doi.org/10.1016/j.whi.2015.03.008

29. Matthews KL. Memorandum: Trauma-Informed Care (TIC) Approach (VIEWS 5592562). Department of Veterans Affairs, Veterans Health Administration; 2021 Aug.

30. Hamberger LK, Barry C, Franco Z. Implementing Trauma-Informed Care in Primary Medical Settings: Evidence-Based Rationale and Approaches. Journal of Aggression, Maltreatment & Trauma. 2019;28:425–44. https://doi.org/10.1080/10926771.2019.1572399

31. Huo Y, Couzner L, Windsor T, Laver K, Dissanayaka NN, Cations M. Barriers and enablers for the implementation of trauma-informed care in healthcare settings: a systematic review. Implement Sci Commun. 2023;4:49. https://doi.org/10.1186/s43058-023-00428-0

32. Carney D. National WH-PBRN Full Community Call: Trauma Informed Care Practice Scan: Results Report. 2021.

33. Woods NF, Hohensee C, Carpenter JS, Cohen L, Ensrud K, Freeman EW, et al. Symptom clusters among MsFLASH clinical trial participants. Menopause. 2016;23:158–65. https://doi.org/10.1097/GME.0000000000000516

34. US Preventive Services Task Force, Mangione CM, Barry MJ, Nicholson WK, Cabana M, Caughey AB, et al. Hormone Therapy for the Primary Prevention of Chronic Conditions in Postmenopausal Persons: US Preventive Services Task Force Recommendation Statement. JAMA. 2022;328:1740. https://doi.org/10.1001/jama.2022.18625

35. The 2022 hormone therapy position statement of The North American Menopause Society. Menopause. 2022;29:767–94. https://doi.org/10.1097/GME.0000000000002028

36. Blanken A, Gibson CJ, Li Y, Huang AJ, Byers AL, Maguen S, et al. Racial/ethnic disparities in the diagnosis and management of menopause symptoms among midlife women veterans. Menopause. 2022;29:877–82. https://doi.org/10.1097/GME.0000000000001978

37. Dietz NA, Mijares-Cantrell T, Acevedo D, Annane D, Rodriguez R, Caralis P, et al. Women veterans and menopause: Knowledge and preferences. Women & Health. 2018;58:898–914. https://doi.org/10.1080/03630242.2017.1363123

38. Barber K, Charles A. Barriers to Accessing Effective Treatment and Support for Menopausal Symptoms: A Qualitative Study Capturing the Behaviours, Beliefs and Experiences of Key Stakeholders. PPA. 2023;Volume 17:2971–80. https://doi.org/10.2147/PPA.S430203

39. Friedman JK, Gibson CJ, Diem S, Fenwick K, Yano EM. Women Veteran Menopause Research. [Internet]. VA Women’s Health Research Network (VA HSR SDR 10-012).; 2024. https://www.hsrd.research.va.gov/centers/womens_health/menopause-snapshot.pdf. Accessed 24 Oct 2024

40. Gibson CJ, Li Y, Jasuja GK, Self KJ, Seal KH, Byers AL. Menopausal Hormone Therapy and Suicide in a National Sample of Midlife and Older Women Veterans. Medical Care. 2021;59:S70–6. https://doi.org/10.1097/MLR.0000000000001433

41. Breland JY, Phibbs CS, Hoggatt KJ, Washington DL, Lee J, Haskell S, et al. The Obesity Epidemic in the Veterans Health Administration: Prevalence Among Key Populations of Women and Men Veterans. J GEN INTERN MED. 2017;32:11–7. https://doi.org/10.1007/s11606-016-3962-1

42. Stefanovics EA, Potenza MN, Pietrzak RH. The physical and mental health burden of obesity in U.S. veterans: Results from the National Health and Resilience in Veterans Study. Journal of Psychiatric Research. 2018;103:112–9. https://doi.org/10.1016/j.jpsychires.2018.05.016

43. Betancourt JA, Stigler Granados P, Pacheco GJ, Shanmugam R, Kruse CS, Fulton LV. Obesity and Morbidity Risk in the U.S. Veteran. Healthcare. 2020;8:191. https://doi.org/10.3390/healthcare8030191

44. Batch BC, Goldstein K, Yancy WS, Sanders LL, Danus S, Grambow SC, et al. Outcome by Gender in the Veterans Health Administration Motivating Overweight/Obese Veterans Everywhere Weight Management Program. Journal of Women’s Health. 2018;27:32–9. https://doi.org/10.1089/jwh.2016.6212

45. Maciejewski ML, Shepherd-Banigan M, Raffa SD, Weidenbacher HJ. Systematic Review of Behavioral Weight Management Program MOVE! for Veterans. American Journal of Preventive Medicine. 2018;54:704–14. https://doi.org/10.1016/j.amepre.2018.01.029

46. Batch BC, Brown CS, Goldstein KM, Danus S, Sperber NR, Bosworth HB. Women Veterans Experience with the VA MOVE! Weight Management Program. Women’s Health Reports. 2020;1:65–72. https://doi.org/10.1089/whr.2019.0009

47. Zhao Z, Serier KN, Smith BN, Vogt D, Kehle-Forbes S, Mitchell KS. Gender similarities and differences in associations between weight discrimination, shape/weight concerns, and eating disorder symptoms among post-9/11 veterans. Eating Behaviors. 2023;51:101818. https://doi.org/10.1016/j.eatbeh.2023.101818
